# Exploring diagnostic language assessment practices in South Africa: A national survey

**DOI:** 10.4102/sajcd.v73i1.1186

**Published:** 2026-09-09

**Authors:** Mignon Botes

**Affiliations:** 1 Department of Speech-Language Pathology, Faculty of Humanities, University of the Witwatersrand, Johannesburg, South Africa

**Keywords:** language assessment, multilingualism, speech-language therapy, diagnostic practices, South Africa, cultural and linguistic diversity

## Abstract

**Background:**

Diagnostic language assessment is central to speech-language therapy; however, in multilingual contexts such as South Africa, clinicians face challenges related to linguistic diversity, limited access to culturally appropriate tools and contextual constraints.

**Objectives:**

This study aimed to describe diagnostic language assessment practices among South African speech-language pathologists (SLPs), including tools used, screening and assessment workflows, domains assessed, time and cost considerations, interpretation practices, and clinician confidence.

**Method:**

A descriptive, cross-sectional survey design was employed. Using convenience and snowball sampling, an online questionnaire was completed by 99 SLPs across South Africa. Data were analysed using descriptive statistics.

**Results:**

Speech-language pathologists reported using a combination of standardised and informal assessment tools, with commonly used instruments including the Renfrew Language Scales, Clinical Evaluation of Language Fundamentals and Test of Auditory Comprehension of Language. Screening practices were inconsistent, and assessments typically spanned one to two sessions. Clinical judgement was the most frequently used interpretive approach, while reliance on standardised norms and monolingual comparisons was less common. Clinicians reported low to moderate confidence when assessing multilingual clients. Key challenges included lack of culturally adapted tools, linguistic bias in assessments and difficulties interpreting bilingual children’s results.

**Conclusion:**

Diagnostic language assessment practices in South Africa reflect adaptive responses to systemic constraints rather than inconsistency in clinician practice.

**Contribution:**

The findings highlight the need for culturally grounded, multilingual assessment frameworks, locally relevant tools and clearer practice guidelines to support equitable diagnostic decision-making.

## Introduction

Diagnostic language assessment is central to speech-language therapy, underpinning the identification of language disorders and guiding intervention planning (Norbury et al., 2016). Clinicians must make complex diagnostic decisions through the integration of multiple sources of information, including standardised test scores, caregiver report, observations and clinical judgement (Pascoe & Singh, 2023). However, diagnostic decision-making is shaped by the nature and quality of available data. Evidence suggests that clinicians demonstrate variability in their judgements, particularly when relying on informal or perceptual measures (Bawayan & Brown, 2022). In South Africa, these challenges are compounded by extensive linguistic diversity and longstanding concerns regarding the validity of assessment tools that have not been developed or normed for multilingual populations (Khoza-Shangase & Mophosho, 2021; Southwood et al., 2025; Van Dulm & Southwood, 2008). A key component of diagnostic practice is the selection and use of assessment tools. In South Africa, speech-language pathologists (SLPs) commonly use standardised instruments such as the Clinical Evaluation of Language Fundamentals (CELF), Peabody Picture Vocabulary Test (PPVT) and Renfrew Action Picture Test, which are primarily developed and normed in Western, monolingual populations (Van Dulm, 2013; Van Dulm & Southwood, 2008). The use of these tools in linguistically diverse populations raises concerns regarding cultural and linguistic validity, as normative samples do not reflect the multilingual realities of South African children (Khoza-Shangase & Mophosho, 2018; Mdlalo, 2016). This mismatch introduces a risk of misdiagnosis, including both over-identification and under-identification of language disorders (Bedore & Peña, 2008; Paradis, 2010). Despite these concerns, standardised assessments continue to play a dominant role in diagnostic decision-making. Globally, clinicians often prioritise standardised test scores when determining eligibility for services (Fulcher-Rood et al., 2018). At the same time, clinicians use informal measures such as language sampling, caregiver interviews and observations to capture functional language use in naturalistic contexts (Verdon et al., 2015). However, these measures are often weighted less heavily in diagnostic decisions and may be applied inconsistently across clinicians (Bawayan & Brown, 2022; Fulcher-Rood et al., 2018), creating a tension between norm-referenced data and more ecologically valid sources of information. This tension may be particularly pronounced in South Africa, where clinicians frequently assess children whose linguistic and cultural backgrounds differ substantially from those represented in available normative datasets. In multilingual contexts such as South Africa, where no single assessment approach adequately captures children’s linguistic repertoires, the complexity of diagnostic assessment has led to recommendations for multi-method approaches. Research emphasises that no single assessment tool is sufficient for diagnosing language disorders, particularly in multilingual populations (Bedore & Peña, 2008). Instead, clinicians integrate multiple data sources to build a comprehensive profile of a child’s language abilities. Approaches such as dynamic assessment have been proposed as more equitable alternatives, as they focus on learning potential rather than static performance (Peña et al., 2001). Assessment practices are shaped by systemic and contextual constraints. South African SLPs often work in resource-constrained environments characterised by high caseloads, limited assessment time and restricted access to culturally appropriate tools (Pascoe & Singh, 2023). Similar challenges have been reported internationally (Hunt et al., 2025), contributing to more streamlined assessment processes and reliance on readily available tools. International evidence further suggests that assessment practices vary across service settings (Fulcher-Rood & Castilla-Earls, 2023), highlighting the importance of understanding how South African clinicians negotiate diagnostic decision-making within their own service contexts. An additional challenge lies in the interpretation of assessment outcomes. Standardised scores are often used as primary indicators of impairment; however, their interpretation is problematic when applied to children whose linguistic and cultural backgrounds differ from those of the normative sample (Carter et al., 2005; Thordardottir, 2015). Clinicians may therefore draw on alternative strategies, including clinical judgement and integration of multiple data sources. Diagnostic decisions typically emerge from the synthesis of diverse and sometimes conflicting information, requiring significant clinical expertise (Hunt et al., 2025; Thomas et al., 2019). Clinician confidence is closely linked to these complexities. In contexts where assessment tools are not well suited to the populations being assessed, clinicians may experience uncertainty in their diagnostic decisions (Khoza-Shangase & Mophosho, 2021). International findings similarly indicate reduced confidence when assessing multilingual children, particularly in the absence of appropriate training and resources (Hunt et al., 2025). Despite growing recognition of the mismatch between available assessment tools and the multilingual realities of South African children, there remains limited empirical evidence describing how South African SLPs conduct diagnostic language assessments in practice. Existing research has largely focused on the mismatch between available tools and linguistic diversity (Mdlalo et al., 2016; Van Dulm, 2013), with less attention given to assessment workflows, including screening practices, areas assessed, time and cost considerations, and decision-making processes. International evidence further suggests that assessment practices may vary across service settings (Fulcher-Rood & Castilla-Earls, 2023). This study aims to provide a comprehensive overview of diagnostic language assessment practices among South African SLPs. Specifically, it examines the tools used, screening and assessment workflows, areas of language assessed, time and resource considerations, scoring and interpretation practices, and clinician confidence in diagnostic decision-making. The study sought to answer the following question: *How do South African SLPs conduct diagnostic language assessments in multilingual and resource-constrained contexts?* By situating these practices within multilingual and resource-constrained contexts, the study contributes to a more contextually grounded understanding of language assessment in South Africa.

## Research methods and design

### Study design and setting

This descriptive, cross-sectional survey investigated language assessment practices in South Africa. Data were collected online using REDCap hosted at the University of the Witwatersrand.

### Study population and sampling

Participants were SLPs registered with the Health Professions Council of South Africa (HPCSA) who conducted language assessments with children. Clinicians across public, private, education and non-governmental organisation (NGO) sectors were eligible. A non-probability convenience sampling strategy, supplemented by snowball sampling, was used to recruit participants. Recruitment was conducted via professional networks and direct email invitations.

### Survey

The questionnaire included demographic items, diagnostic language assessment practices and language assessment adaptations. Only diagnostic practice items are reported here. Questions covered screening, tools used, domains assessed, assessment time, cost, scoring, interpretation and clinician confidence. A pilot with four SLPs resulted in minor wording refinements.

### Data collection

Participants provided electronic consent before completing the survey.

### Data analysis

Data were cleaned and analysed using Microsoft Excel. Responses with 100% completion were included. Descriptive statistics summarised categorical and numerical variables.

### Ethical considerations

Ethical clearance to conduct this study was obtained from the University of the Witwatersrand Human Research Ethics Committee (No. H24/09/05). Participation was voluntary and anonymous.

## Results

### Participant characteristics

Consistent with the study aim of describing the characteristics of clinicians undertaking diagnostic language assessment in South Africa, 99 SLPs completed the full survey. Participants represented all nine provinces, with the largest proportion practising in Gauteng (*n* = 56, 56.6%), followed by the Western Cape (*n* = 16, 16.2%) and KwaZulu-Natal (*n* = 10, 10.1%). Nearly half of the participants worked in private practice (*n* = 47, 47.5%), followed by remedial schools (*n* = 21, 21.2%) and government institutions (*n* = 14, 14.1%). Participants represented a broad range of clinical experience, with the largest group reporting more than 21 years of practice (*n* = 32, 32.3%) (Table 1).

**TABLE 1:** Participant demographics (*N* = 99).

Variable	*n*	%
**Province**		
Gauteng	56	56.6
Western Cape	16	16.2
KwaZulu-Natal	10	10.1
Eastern Cape	8	8.1
Free State	3	3
North West	2	2
Northern Cape	2	2
Limpopo	1	1
Mpumalanga	1	1
**Race**		
White people	77	77.8
Indian and/or Asian people	10	10.1
Coloured people	7	7.1
Black people	5	5.1
**Years practising**		
0–3	18	18.2
4–7	14	14.1
8–15	19	19.2
16–20	16	16.2
21+	32	32.3
**Primary workplace**		
Private practice	47	47.5
Remedial school	21	21.2
Government institution	14	14.1
Hospital	6	6.1
Other	11	11.1

### Clinical and linguistic practice profile

To characterise the contexts in which diagnostic language assessments are conducted, most SLPs reported English as their primary language of practice (*n* = 79), although they routinely assessed children across multiple South African languages, particularly Afrikaans (*n* = 58), isiZulu (*n* = 34) and isiXhosa (*n* = 27). Language delay (*n* = 77), autism spectrum disorder (*n* = 51) and speech sound disorders (*n* = 49) were the most frequently encountered diagnoses. Receptive (*n* = 99%) and expressive language (*n* = 98) were the domains most consistently assessed during diagnostic evaluations (Table 2).

**TABLE 2:** Clinical and linguistic practice profile and domains assessed (*N* = 99).

Variable	*n*	%
**Primary language of practice**		
English	79	79.8
Afrikaans	16	16.2
Other SA languages	4	4.0
**Languages commonly encountered†**		
Afrikaans	58	-
English	29	-
isiZulu	34	-
isiXhosa	27	-
Sesotho	20	-
Sepedi	14	-
Other African languages	14	-
**Common diagnoses†**		
Language delay	77	-
ASD	51	-
Speech sound disorders	49	-
APD	35	-
Literacy delays	23	-
Neurological and/or genetic disorders	21	-
Aural rehabilitation	3	-
**Domains assessed in diagnostics†**		
Receptive language	99	-
Expressive language	98	-
Speech sound development	94	-
Oral mechanism exam	93	-
Pragmatics	72	-
Phonological awareness	82	-
Auditory processing	82	-
Reading	59	-
Spelling and/or writing	60	-
Other	17	-

SA, South Africa; ASD, Autism Spectrum Disorder; APD, Auditory Processing Disorder.

† , Multiple responses allowed.

### Screening practices

To address the study aim relating to screening workflows, screening practices varied considerably. Twenty-eight clinicians (28.3%) reported routinely using a standardised screener, while 43 (43.4%) indicated that they sometimes used one. In contrast, 60 clinicians (60.6%) reported using self-developed screening tools. Fifty-one clinicians (51.5%) indicated that they did not always screen before conducting a full diagnostic assessment, whereas only 12 (12.1%) reported consistently screening before assessment. Screening informed subsequent diagnostic decision-making for 59 participants (59.6%) (Table 3).

**TABLE 3:** Screening practices (*N* = 99).

Screening practice	Yes		Sometimes		No		Not applicable	
	*n*	%	*n*	%	*n*	%	*n*	%
Use a standardised screener	28	28.3	43	43.4	27	27.3	1	1.0
Use a self-developed screener	60	60.6	16	16.2	14	14.1	9	9.1
Screening informs diagnostics	59	59.65	28	28.3	4	4.0	7	7.1
Screening only before starting intervention (no diagnostic assessment)	7	7.1	36	36.4	54	54.5	2	2.0
Always screen before full diagnostic assessment	12	12.1	33	33.3	51	51.5	3	3.0

### Assessment tools and interpretation practices

To describe diagnostic assessment practices, SLPs reported using a combination of standardised and informal assessment tools. The most commonly used standardised assessments were the Renfrew Language Scales (*n* = 77), the Test of Auditory Comprehension of Language (TACL) (*n* = 69) and the CELF (*n* = 63). Informal speech assessments were also widely used (*n* = 67). Clinical judgement was the most frequently reported interpretation strategy (*n* = 84), followed by consideration of bilingual language development (*n* = 38) and the use of standardised norms (*n* = 35). Comparison with monolingual peers was reported by only 10 clinicians (Table 4).

**TABLE 4:** Tools used and interpretation practices (*N* = 99).

Variable	*n*
**Assessment tools used†**	
Renfrew Language Scales	77
Clinical Evaluation of Language Fundamentals (CELF)	63
The Test of Auditory Comprehension of Language (TACL)	69
Informal speech assessments	67
Test of Language Development (TOLD)	36
Preschool Language Scales (PLS)	28
Peabody Picture Vocabulary Test (PPVT)	14
New Reynell Developmental Language Scales (NRDLS)	14
Comprehensive Assessment of Spoken Language (CASL)	6
**Interpretation practice†**	
Clinical judgement	84
Consider bilingual development	38
Use standardised norms	35
Compare to monolingual peers	10
Other	4

† , Multiple responses allowed.

### Assessment duration, sessions and cost

To describe the logistics of diagnostic language assessment, assessments most commonly lasted between 1.5 h and 2 h (*n* = 36, 36.4%) or between 1 h and 1.5 h (*n* = 31, 31.3%). Assessments were most frequently conducted over two sessions (*n* = 41, 41.4%), followed by one session (*n* = 34, 34.3%). Costs varied according to service setting, with 37 clinicians (37.4%) indicating that fees were not applicable, while private assessment fees most commonly ranged between R1000.00 and R1500.00 (*n* = 26, 26.3%) (Table 5).

**TABLE 5:** Assessment duration, number of sessions and cost (*N* = 99).

Assessment variable	*n*	%
**Duration**		
45 min – 1 h	18	18.2
1 h – 1 h 30 min	31	31.3
1 h 30 min – 2 h	36	36.4
More than 2 h	14	14.1
**Number of sessions**		
One session	34	34.3
Two sessions	41	14.4
Three sessions	24	24.2
**Cost**		
Not applicable	37	37.4
R1000.00 – R1500.00	26	26.3
R1500.00 – R2000.00	12	12.1
R2000.00 – R2500.00	15	15.2
R2500.00 – R3000.00	5	5.1
> R3000.00	4	4.0

### Clinician confidence and challenges

To address the study aim relating to clinician confidence and barriers to diagnostic assessment, most participants reported being somewhat confident (*n* = 38, 38.4%) or neutral (*n* = 25, 25.3%) when assessing multilingual children, while only six clinicians (6.1%) reported being very or extremely confident. The most frequently reported challenges were the lack of culturally adapted assessment tools (*n* = 71), the high cost of assessment materials (*n* = 65), difficulties translating assessment materials (*n* = 54) and interpreting bilingual children’s performance (*n* = 54) (Table 6).

**TABLE 6:** Clinician confidence and challenges (*N* = 99).

Variable	*n*	%
**Confidence level**		
Extremely confident	1	1.0
Very confident	5	5.1
Confident	10	10.1
Somewhat confident	38	38.4
Neutral	25	25.3
Not confident	19	19.2
**Challenges†**		
Lack of culturally adapted tests	71	-
Issues translating materials	54	-
Interpreting bilingual children	54	-
High cost of tools	65	-
Difficulty obtaining materials	37	-
Linguistic bias	44	-
Cultural communication differences	49	-
Lack of norms	48	-
Lack of multilingual support	35	-
Limited access to tests	33	-
Time to adapt tests	24	-
Lack of training	24	-
Parents misunderstanding results	21	-
Other	2	-

† , Multiple responses allowed.

## Discussion

The findings of this study illustrate a diagnostic landscape characterised by variability, fragmentation and professional adaptation, reflecting the structural realities of practising in a linguistically diverse and resource-constrained country. These patterns reflect systemic gaps in the availability and cultural relevance of diagnostic tools in South Africa (Pascoe & Singh, 2023; Salako, 2025; Southwood et al., 2025).

A central interpretive insight is that SLPs are navigating two competing demands: the requirement to provide accurate, defensible diagnostic decisions and the lack of validated, culturally fair tools. The high reliance on tools such as the CELF, TACL and Renfrew tasks – none of which are normed on South African populations – reflects the absence of viable, locally normed alternatives. This suggests that SLPs are working within a diagnostic ecology shaped by historical inequities in the development of assessment tools and the dominance of monolingual English norms (Pascoe & Singh, 2023; Van Dulm, 2013; Van Dulm & Southwood, 2008). This reflects broader structural constraints identified across African contexts (Salako, 2025). The pervasive use of informal measures and clinician-developed tools reflects adaptive, context-responsive practice. Speech-language pathologists appear to draw on linguistically attuned approaches such as narrative assessment, observation and language sampling, which align more closely with multilingual development than standardised tools (Higgins & Lefebvre, 2024; Verdon et al., 2015). However, informal measures may introduce variability when not supported by systematic frameworks (Bawayan & Brown, 2022). Moderate clinician confidence reflects the limits of relying on individual expertise within constrained systems (Thomas et al., 2019). The screening practices reveal a similar tension. Screening practices varied, reflecting tension between standardisation and contextual responsiveness. Speech-language pathologists appear to favour flexible approaches, suggesting that imported screening models may not align with multilingual contexts. This reflects broader concerns regarding the cultural validity of standardised frameworks (Bedore & Peña, 2008; Carter et al., 2005). Interpretation practices provide an important insight into how clinicians navigate diagnostic uncertainty in multilingual contexts. Rather than representing a weakness, the dominance of clinical judgement may reflect an appropriate and necessary strategy when standardised measures are not culturally or linguistically valid. Multilingual development, variable language exposure and cultural communication norms challenge traditional psychometric models (Bedore & Peña, 2008; Bortz, 2023; Paradis et al., 2021), requiring clinicians to integrate information from multiple sources rather than rely on test scores alone. The low use of monolingual comparison groups further suggests awareness of their limited validity and supports recommendations that diagnostic decisions should be based on the synthesis of standardised measures, language samples, caregiver report, observations and informed clinical judgement (Thomas et al., 2019; Verdon et al., 2015). The challenges identified by clinicians – particularly linguistic bias, a lack of adapted tools and difficulties in interpreting bilingual children’s results – reflect interconnected systemic inequities. These findings support arguments regarding the Eurocentricity of Speech-Language and Hearing training and tools (Khoza-Shangase & Mophosho, 2018, 2021). Finally, the data provide insight into the relationship between multilingualism and diagnostic certainty. Clinician confidence was generally modest, reflecting broader systemic constraints. Similar patterns have been reported in South African contexts (Mdlalo et al., 2016; Pascoe et al., 2010). Taken together, these findings indicate that improving diagnostic language assessment in South Africa will require more than the development of new tools. It will necessitate a broader reframing of assessment theory, training and policy to recognise multilingualism as the norm. This aligns with broader calls for culturally grounded, contextually responsive frameworks (Salako, 2025).

### Limitations

This study relied on self-reported data, reflecting clinicians’ perceptions rather than direct observation of practice. Although this limits conclusions about actual clinical behaviour, these perceptions provide valuable insight into how diagnostic decisions are experienced within structurally constrained multilingual contexts. While geographically diverse, the sample may underrepresent SLPs outside established professional networks. The survey captured broad patterns of practice but not the detailed reasoning underlying diagnostic decisions. In addition, as this article reports only the diagnostic component of a larger study, insights into adaptation processes are presented elsewhere. These limitations should therefore be considered when interpreting the findings, which are intended to describe national practice patterns rather than establish causal relationships.

### Strengths

This study provides a national snapshot of diagnostic language assessment practices within a multilingual context. By examining tool use, screening, interpretation and clinician confidence, it offers empirical evidence of how systemic constraints shape practice. The findings move beyond anecdotal accounts to demonstrate the gap between available assessment models and local linguistic realities, providing evidence to inform clinician training, policy development and future assessment initiatives. Importantly, the study demonstrates that multilingualism is not a peripheral consideration but a defining feature of diagnostic language assessment in South Africa, with implications for how assessment practices, guidelines and tools are conceptualised and implemented.

### Implications for practice and policy

The findings indicate a misalignment between current assessment models and the linguistic and cultural profiles of South African clients. Strengthening practice requires more than increased access to tools; it necessitates a shift toward multilingual, culturally responsive frameworks. Priorities include the development of locally relevant assessment tools, clearer guidelines supporting dynamic and contextually informed approaches, and recognition of the role of informed clinical judgement where standardised options are limited. Addressing cost and access barriers to assessment materials is also essential for equitable service delivery.

### Future research

Future research should focus on developing and validating culturally grounded frameworks for multilingual language assessment. Studies are needed to evaluate the validity of informal and clinician-developed tools currently used in practice. Further work should explore clinicians’ decision-making processes and examine how multilingual exposure influences assessment outcomes. Collaborative research with communities will be critical to ensure that assessment approaches reflect local languages, cultures and contexts.

## Conclusion

This study reveals a diagnostic landscape characterised not by inconsistency, but by clinicians’ adaptive responses to systemic constraints and the enduring inadequacy of monolingual assessment models in a multilingual society. SLPs in South Africa navigate a complex intersection of linguistic diversity, limited access to culturally valid tools and structural inequities that shape both their assessment choices and their diagnostic confidence. The findings underscore the need for a fundamental reorientation of assessment theory, training and policy – towards culturally grounded, multilingual frameworks (International Expert Panel, 2017) that reflect the realities of South African clients. Achieving equitable diagnostic language assessment will require sustained investment in the development, validation and implementation of locally relevant assessment tools and multilingual assessment practices. Meaningful progress will depend not only on creating new tools, but also on transforming the epistemological foundations of diagnostic language assessment to recognise multilingualism as central to, rather than peripheral in, clinical practice.
